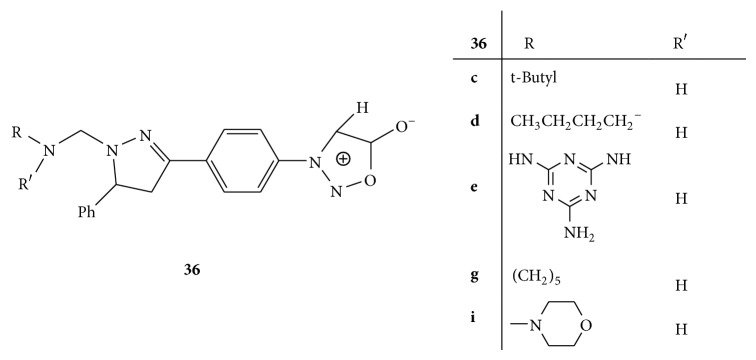# Mannich Bases: An Important Pharmacophore in Present Scenario

**DOI:** 10.1155/2014/191072

**Published:** 2014-11-12

**Authors:** Suman Bala, Neha Sharma, Anu Kajal, Sunil Kamboj, Vipin Saini

**Affiliations:** M. M. College of Pharmacy, Maharishi Markandeshwar University, Mullana, Ambala, Haryana 133207, India

## Abstract

Mannich bases are the end products of Mannich reaction and are known as beta-amino ketone carrying compounds. Mannich reaction is a carbon-carbon bond forming nucleophilic addition reaction and is a key step in synthesis of a wide variety of natural products, pharmaceuticals, and so forth. Mannich reaction is important for the construction of nitrogen containing compounds. There is a number of aminoalkyl chain bearing Mannich bases like fluoxetine, atropine, ethacrynic acid, trihexyphenidyl, and so forth with high curative value. The literature studies enlighten the fact that Mannich bases are very reactive and recognized to possess potent diverse activities like anti-inflammatory, anticancer, antifilarial, antibacterial, antifungal, anticonvulsant, anthelmintic, antitubercular, analgesic, anti-HIV, antimalarial, antipsychotic, antiviral activities and so forth. The biological activity of Mannich bases is mainly attributed to *α*, *β*-unsaturated ketone which can be generated by deamination of hydrogen atom of the amine group.

## 1. Introduction

**Scheme 1:**
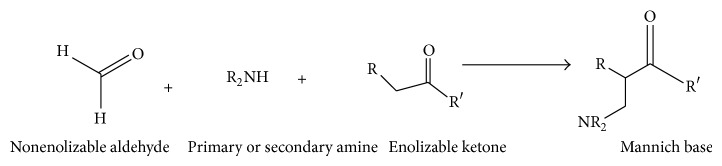


Mannich bases, beta-amino ketones carrying compounds, are the end products of Mannich reaction [1, 2]. Mannich reaction is a nucleophilic addition reaction which involves the condensation of a compound with active hydrogen(s) with an amine (primary or secondary) and formaldehyde (any aldehyde) [3]. The schematic representation of general Mannich reaction is given in Scheme 1.

Mannich bases also act as important pharmacophores or bioactive leads which are further used for synthesis of various potential agents of high medicinal value which possess aminoalkyl chain. The examples of clinically useful Mannich bases which consist of aminoalkyl chain are cocaine, fluoxetine, atropine, ethacrynic acid, trihexyphenidyl, procyclidine, ranitidine, biperiden [4–6], and so forth. Mannich bases are known to play a vital role in the development of synthetic pharmaceutical chemistry. The literature studies revealed that Mannich bases are very reactive and can be easily converted to other compounds, for example, reduced to form physiologically active amino alcohols [7]. Mannich bases are known to possess potent activities like anti-inflammatory [8, 9], anticancer [10, 11], antifilarial [8], antibacterial [12, 13], antifungal [13, 14], anticonvulsant [15], anthelmintic [16], antitubercular [17, 18], analgesic [19], anti-HIV [17], antimalarial [20], antipsychotic [21], antiviral [22] activities and so forth. Along with biological activities Mannich bases are also known for their uses in detergent additives [23], resins, polymers, surface active agents [24], and so forth. Prodrugs of Mannich bases of various active compounds have been prepared to overcome the limitations [25]. Mannich bases (optically pure chiral) of 2-naphthol are employed for catalysis (ligand accelerated and metal mediated) of the enantioselective carbon-carbon bond formation. Mannich bases and their derivatives are intermediates for the synthesis of bioactive molecules [26, 27]. Mannich reaction is widely used for the construction of nitrogen containing compounds [28]. Mannich bases have gained importance due to their application in antibacterial activity [29] and other applications are in agrochemicals such as plant growth regulators.

## 2. Biological Activities

### 2.1. Antimicrobial Activity

**Figure 1:**
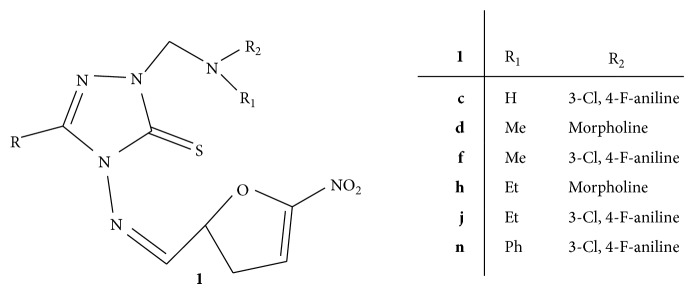


A novel series of Mannich bases of 3-substituted-4-(5-nitro-2-furfurylidene) amino-5-mercapto-1,2,4-triazoles** 1(a–n)** (Figure 1) was synthesized and screened for the antifungal activity against* C. albicans* by employing disc diffusion method using nitrofurazone and fluconazole as standard drugs for comparison. The results revealed that all the compounds were found to be least active as compared to nitrofurazone whereas compounds** 1c**,** 1d**,** 1f**,** 1h**,** 1j**, and** 1n** were found to have better antifungal activity as compared to fluconazole. Compound** 1f** substituted with methyl and chloro group has shown highest antifungal activity with 17 mm zone of inhibition which is higher than zone of inhibition of fluconazole, that is, 12 mm. These results enlighten the key role of chloro group towards antifungal activity [30].

**Figure 2:**
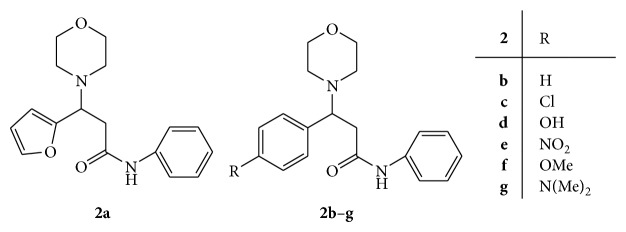


Mannich bases of 2-(phenyl)-2-(morpholine-4-yl)-N-phenylacetamide** 2(a–g)** were synthesized (Figure 2) and screened for antimicrobial activity against various bacterial and fungal strains. Ciprofloxacin and clotrimazole were used as standard drugs for antibacterial and antifungal activities, respectively. From the synthesized compounds,** 2c**, 3-(4-chlorophenyl)-3-(morpholin-4-yl)-*N*-phenylpropanamide, has shown highest antibacterial activity against* S. epidermidis* with 20 mm of zone of inhibition as compared to ciprofloxacin with 15 mm. Compounds** 2e**, 3-(morpholin-4-yl)-3-(4-nitrophenyl)-*N*-phenylpropanamide, and** 2f**, 3-(4-methoxyphenyl)-3-(morpholin-4-yl)-*N*-phenylpropanamide, were found to have equipotent antibacterial activity as compared to ciprofloxacin against* K. pneumonia* and nonhemolytic* streptococcus*, respectively. The compounds** 2d** and** 2e** were found to have equipotent antifungal activity against* M. audouinii* and* C. albicans* as compared to clotrimazole. The compound** 2c** having 4-chlorophenyl has not contributed to antifungal activity [31].

**Figure 3:**
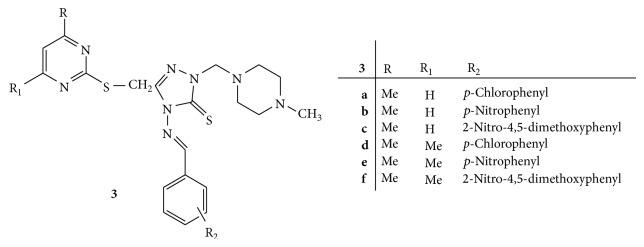


A series of novel Mannich bases of 3-(4,6-disubstituted-2-thiomethylpyrimidyl)-4-amino-5-mercapto-1,2-4-triazoles** 3(a–f)** (Figure 3) was synthesized and further subjected to antibacterial activity against* P. aeruginosa*,* S. marcescens*,* S. aureus*, and* E. coli* [32].

All the synthesized compounds have shown good antibacterial activity against* P. aeruginosa* whereas they were found to be less potent against* S. marcescens*,* S. aureus*, and* E. coli* when compared with standard drug tetracycline. Compounds** 3b**,** 3c,** and** 3e** substituted with nitro group were found to be highly active among all the synthesized compounds [32].

**Figure 4:**
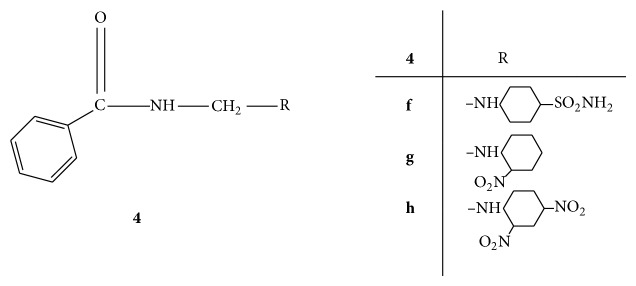


A new series of benzamide substituted Mannich bases** 4(a–g)** (Figure 4) were synthesized and evaluated for antibacterial activity against* E. coli, P. aeruginosa, E. faecalis*, and* S. aureus*. Test tube dilution method was employed for evaluation by using amoxicillin and cefixime as standard drugs for comparison. The results were recorded in terms of minimum inhibitory concentration (MIC). The compounds** 4e**,** 4f**, and** 4g** were found to be highly active among all the synthesized compounds with comparable MIC values of both standard drugs amoxicillin (1.56, 1.56, 3.125, and 3.125) and cefixime (6.25, 6.25, 12.5, and 6.25). The compound** 4g** was found to be most active with MIC (3.125, 3.125, 3.125, and 6.25). These results ensured that substitutions with sulphonamido (**4e)**,* p-*nitro (**4f**), and dinitro (**4g**) groups enhance the antibacterial activity [33].

**Figure 5:**
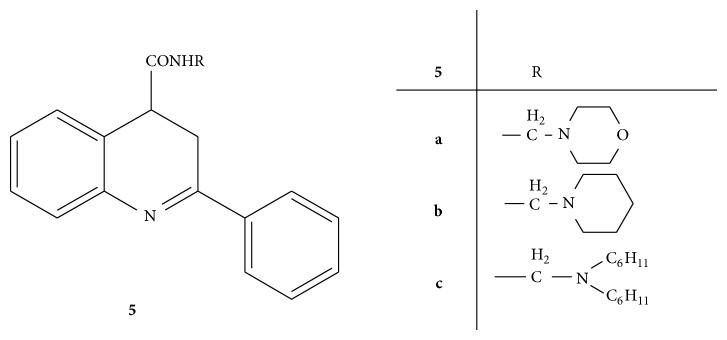


A novel series of Mannich bases** 5(a–e)** (Figure 5) of quinoline derivative (cinchophen) was carried out and screened for the antimicrobial activity against various bacterial and fungal strains. Norfloxacin and fluconazole were used as standard drugs for antibacterial and antifungal activities, respectively. From the synthesized compounds,** 5a**,** 5b**, and** 5c** substituted with morpholine, piperidine, and dicyclohexylamine, respectively, have emerged out as more potent antimicrobial agents than cinchophen and both of the standard drugs. The compounds** 5d** and** 5e** were almost equipotent to cinchophen but did not respond to fungal strains [34].

**Figure 6:**
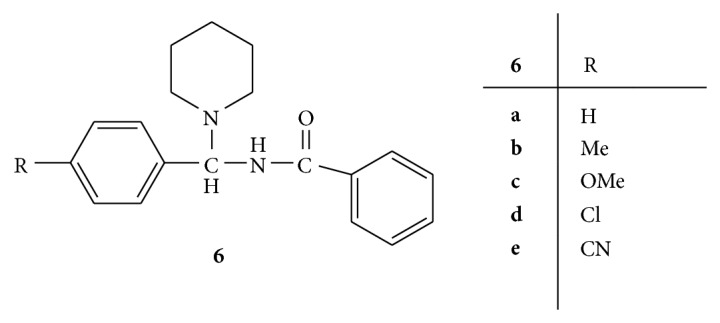


A series of various substituted N-[(1-piperidinobenzyl)benzamide] (PBB)** 6(a–e)** (Figure 6) have been synthesized. The antimicrobial activity of synthesized PBB was carried out on the microorganisms* S*.* aureus, B. subtilis, E. coli*, and* P. aeruginosa* by well diffusion method using ampicillin as standard drug. It has been found that all the compounds were found to be more active against all bacterial strains than ampicillin whereas compounds** 6d** and** 6e** substituted with chloro and cyano group were found to most potent. There results revealed that introduction of electron withdrawing groups on the phenyl ring enhances the activity whereas electron releasing groups are less active compared to unsubstituted phenyl ring [35].

**Figure 7:**
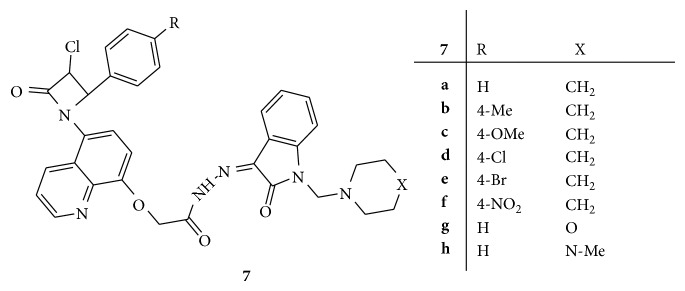


Synthesis of novel Mannich bases (Z)-2-(5-(3-chloro-2-oxo-4-*p*-tolylazetidin-1-yl)quinolin-8-yloxy)-*N*′-(2-oxo-1-(piperidin-1-ylmethyl)indolin-3-ylidine)acetohydrazides,** 7(a–h)**, (Figure 7) was carried out and screened for antibacterial activity against* S. aureus, B. cereus, E. coli*, and* P. aeruginosa*. Amoxicillin and cefaclor were used as reference drugs. Among all the synthesized compounds** 7d**,** 7e,** and** 7f** were found to be highly active. These results suggested that substitution with the nitro, chloro, and bromo group has led to enhanced antibacterial activity [36].

**Figure 8:**
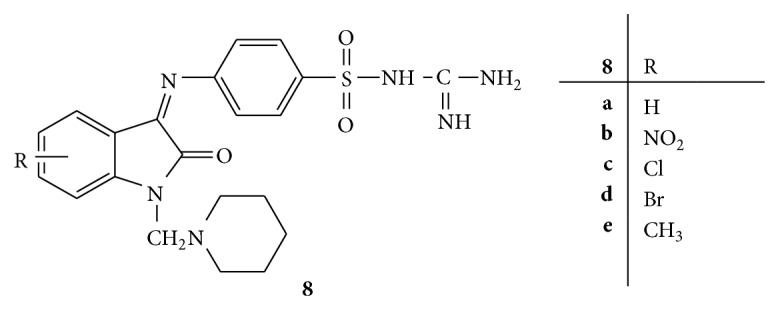


A series of N-Mannich bases of isatin** 8(a–e)** were prepared with 4-amino-*N*-carbamimidoyl benzene sulphonamide (Figure 8). The synthesized compounds were then screened for antibacterial activity and 4-amino-*N*-carbamimidoyl benzene sulphonamide was used as reference drug. From the antibacterial screening it was found that the compound bearing chloro group at 5-position has prominent activity against all the bacterial strains with lowest MIC values [37].

**Figure 9:**
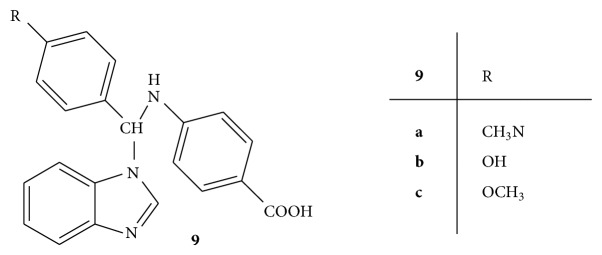


A series of 4-[1H-benzimidazole-yl(substituted benzal)methyl-amino] benzoic acids** 9(a–c)** (Figure 9) was synthesized and screened for the antibacterial and antifungal activities against* S. aureus*,* B. subtilis*,* S. typhi*,* E. coli*,* A. niger*, and* C. albicans*, respectively. Ciprofloxacin and ketoconazole were used as standard drugs for antibacterial and antifungal activities, respectively. The results were obtained in terms of MIC values. The compounds** 9b** and** 9c** substituted with hydroxyl and methoxy group on aromatic ring have shown the highest antibacterial activity whereas compound** 9a** substituted with dimethyl amine has shown highest antifungal activity [38].

**Figure 10:**
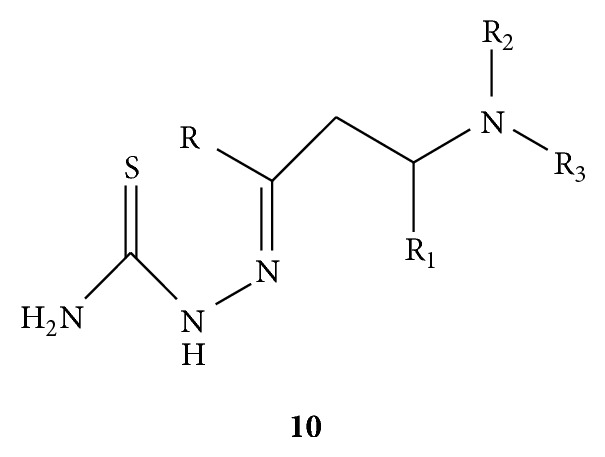


Mannich bases of thiosemicarbazide** 10(a–y)** (Figure 10) were synthesized and evaluated for the antifungal activity against* C. albicans* and* A. niger* by using brain heart infusion (BHI) method to estimate the MIC. Fluconazole was employed as standard drug for comparison. From the results it was concluded that analogs with only alkyl groups have shown comparable activity for both strains as compared to fluconazole whereas acyl derivatives were found to be partly active. Analogs with aromatic and substituted aromatic aldehydes showed least activity, while analogs with aliphatic aldehyde, ketones, and amines showed greater activity in* C. albicans* compared to* A. niger*. The analogs substituted with morpholine and aromatic ketone were found to highly active. Compounds** 10(q–t)** have shown comparable highest activities [39].

**Figure 11:**
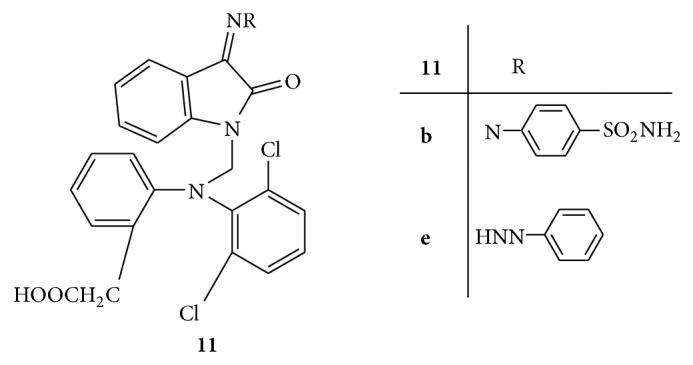


A series of Mannich bases of isatin and its derivatives with 2-[2,6-dichlorophenyl)amino] phenylacetic acid** 11(a–f)** (Figure 11) was synthesized. All the derivatives were evaluated for antibacterial and antifungal activities. The results configured that all the synthesized compounds have shown moderate antibacterial activity except** 11b**. The reason for moderate activity may be due to steric hindrance in binding of bacteria caused by bulky phenyl acetic acid group present at nitrogen atom of isatin nucleus. The compound** 11b** has shown good activity against all the bacteria indicating that the substitution of isatin at the 3-position with a substituted phenyl ring favors antibacterial activity compared to an unsubstituted phenyl ring at the same position. All the compounds have shown significant antifungal activity, especially compound** 11e**, indicating that unsubstituted phenyl ring at 3-position of isatin is desired for antifungal activity compared to substituted phenyl ring [40].

**Figure 12:**
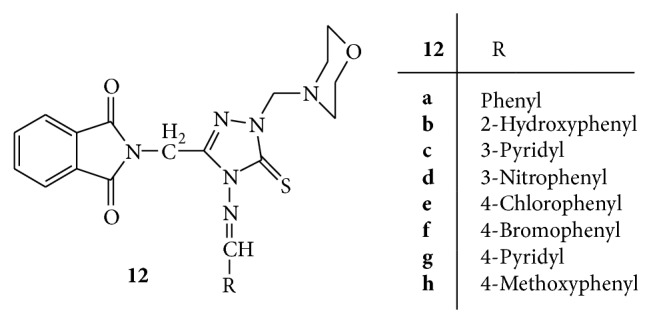


A series of novel Mannich bases** 12(a–h)** of 4-amino-3-(N-phthalimido-methyl)-1,2,4-triazole-5-thione (Figure 12) was synthesized and evaluated for the antimicrobial activity against a variety of gram positive, gram negative, and fungal strains. Levofloxacin was used as standard drug. The results revealed that Mannich bases** 12h** with electron donating substituents on phenyl ring showed comparable activity to levofloxacin against* E. coli* and* K. pneumonia*. Mannich bases** 12e** and** 12f** with halogens on phenyl ring have shown activity very close to levofloxacin against* P. aeruginosa*. Mannich base** 12b** with 2-hydroxy group has shown highest antifungal activity against* C. albicans*. The results also enlightened the fact that the presence of morpholine ring in heterocyclic molecules increases the antimicrobial activity [41].

### 2.2. Anti-Inflammatory Activity

**Figure 13:**
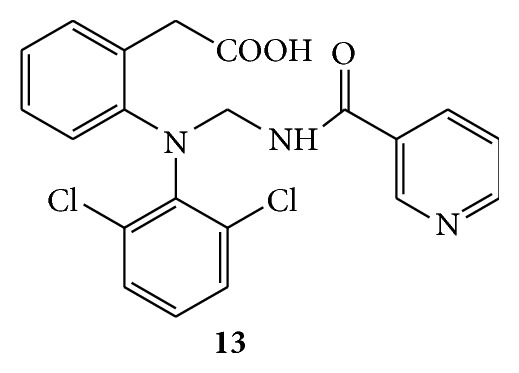


**Figure 14:**
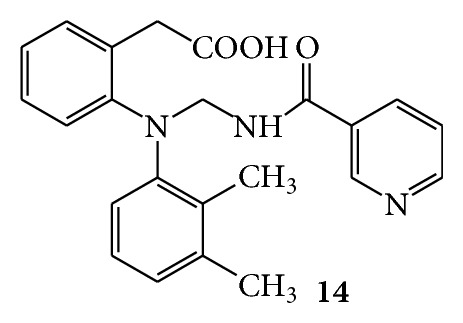


Mannich bases of nicotinamide were synthesized with secondary amines like mefenamic acid,** 13** (Figure 13), and diclofenac,** 14** (Figure 14), in the presence of formaldehyde and hydrochloric acid. Anti-inflammatory activity was studied by hind paw oedema method using carrageenan as a phlogistic agent on Wistar rats. Nicotinamide, diclofenac, and mefenamic acid were used as standard drugs. Both the Mannich bases showed greater anti-inflammatory activity than the corresponding parent drugs [42].

Nicotinamide showed least anti-inflammatory activity. Mannich bases of diclofenac (MND) showed greater activity than Mannich bases of mefenamic acid (MNM). The order of anti-inflammatory activity was found as MND > MNM > diclofenac > mefenamic acid > nicotinamide. It appears that the synthesized Mannich bases showed synergistic anti-inflammatory activity [42].

**Figure 15:**
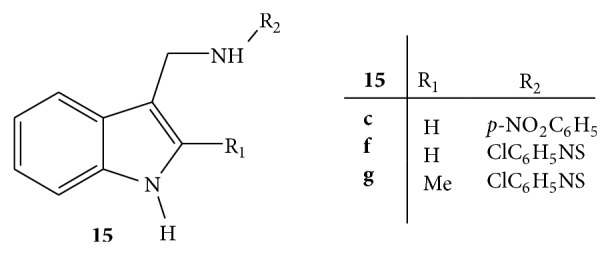


Mannich bases of indole,** 15,** derivatives were synthesized by reacting different derivatives of indole with various aromatic and heterocyclic amines in the presence of formaldehyde and dimethylformamide (Figure 15). Anti-inflammatory activity was screened on albino rats of Wistar strains by carrageenan paw induced method. Diclofenac sodium was used as standard drug. Carrageenan suspension 0.9% in sodium chloride was injected in plantar region of hind paw and paw volume was measured with the aid of plethysmometer. It was observed that the newly synthesized Mannich bases** 15c**,** 15f,** and** 15g** possessing electron withdrawing groups (nitro and chloro) exhibited better anti-inflammatory activity [43].

**Figure 16:**
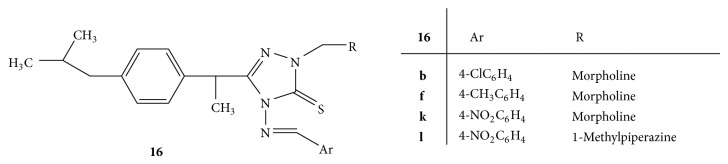


A series of 4-[(4-aryl) methylidene] amino-2-(substituted-4-ylmethyl)-5-{1-[4-(2-methylpropyl)phenyl]ethyl}-2,4-dihydro-3H-1,2,4-triazole-3-thiones** 16**(**a–n**) (Figure 16) was synthesized from ibuprofen by a three-component Mannich reaction. The synthesized Mannich bases were screened for their anti-inflammatory activity by using carrageenan induced rat paw oedema model. Diclofenac and ibuprofen were used as standard drug substances. The Mannich bases** 16b**,** 16f**,** 16k**, and** 16l** carrying morpholino and* N*-methylpiperazino residues were found to be most promising anti-inflammatory agents [44].

**Figure 17:**
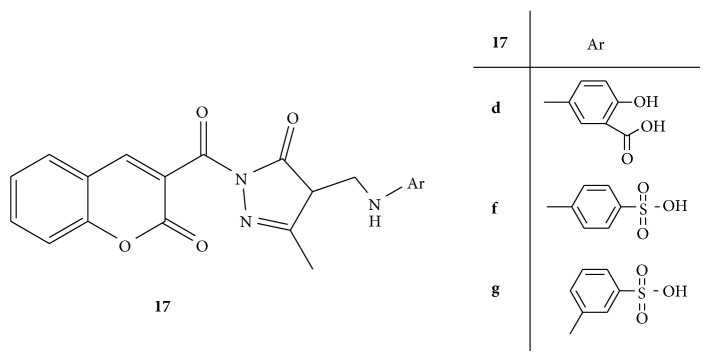


A series of Mannich bases of 5-methyl-2-[(2-oxo-2H-chromen-3-yl)carbonyl]-2,4-dihydro-3H-pyrazol-3-one** 17**(**a–j**) (Figure 17) was synthesized by using conventional and nonconventional (microwave) techniques. The newly synthesized Mannich bases were screened for their anti-inflammatory activity by means of carrageenan induced rat paw oedema model. Indomethacin was used as standard drug substance for comparison of results. Among all the compounds,** 17f** and** 17g** containing sulfonic group at* para* and* meta* positions, respectively, have shown potent anti-inflammatory activity along with minimum ulcerogenic index. In the compound** 17d** introduction of bulky group resulted in the extreme decrease in the activity [45].

### 2.3. Anthelmintic Activity

**Figure 18:**
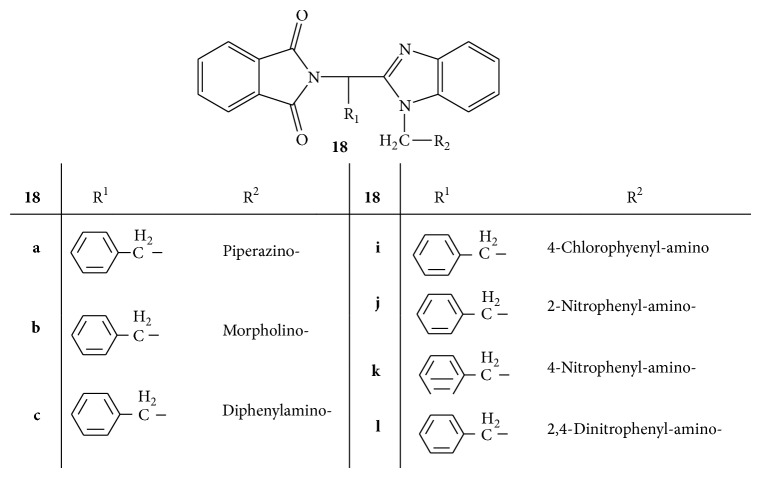


*N*-Mannich bases of benzimidazolyl substituted 1H-isoindole-1,3(2H)-dione** 18(a–l)** were synthesized (Figure 18). All the synthesized compounds were screened for anthelmintic activity by Watkins technique, against common Indian earthworm* P. posthuma*. Piperazine hydrochloride was used as standard drug. All the synthesized derivatives have shown significant anthelmintic activity whereas the derivatives substituted with different groups, that is,** 18a** (piperazino-),** 18b** (morpholine-),** 18c** (diphenylamino-),** 18i** (chloro), and** 18j–l** (nitro and dinitro) groups, showed better activity than other derivatives [46].

**Figure 19:**
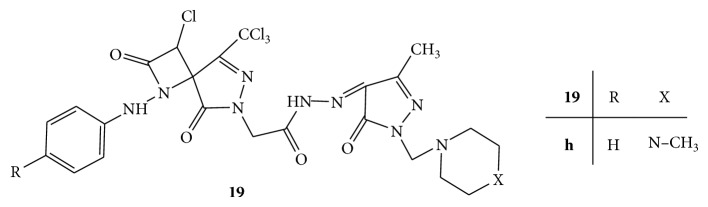


A novel series of eight Mannich bases, 2-(3-chloro-2,5-dioxo-1-(4-substituted phenylamino)-8-(trichloromethyl)-1,6,7-triazaspiro[3.4]oct-7-en-6-yl)-N′-((3-methyl-5-oxo-1-(morpholine/piperidin/N-methylpiperazine-1-ylmethyl)-4,5-dihydro-1H-pyrazol-4-yl)methylene)acetohydazides** 19(a–h)** (Figure 19) were synthesized. All the synthesized Mannich bases were evaluated for their anthelmintic activity against* P. posthuma*. Piperazine citrate was used as standard drug. The compound** 19h** containing N-methylpiperazine moiety was found to have significant anthelmintic activity [47].

### 2.4. Anticonvulsant Activity

**Figure 20:**
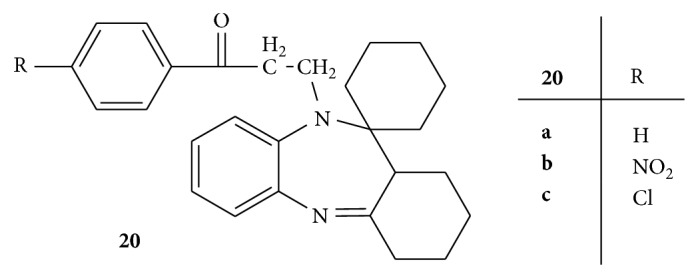


**Figure 21:**
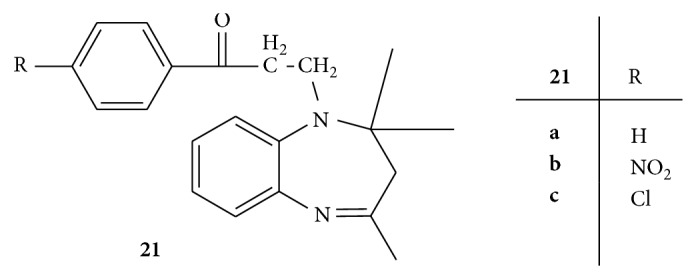


Synthesis of two series,** 20**,** 21(a–c),** of Mannich bases of 1,5-benzodiazepines was carried out and evaluated for the anticonvulsant activity by isoniazid and thiosemicarbazide induced convulsion model (Figures 20 and 21). Among all the synthesized compounds** 20a** with cyclohexane at position 2 and** 21a** with methyl group at positions 2 and 4 were found to most active among all the synthesized compounds. The compounds** 20b**,** 20b**, and** 21c** were found to be least active [48].

**Figure 22:**
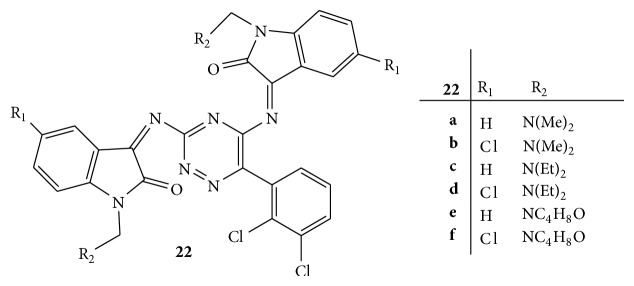


A series of various Mannich base derivatives of lamotrigine,** 22(a–f),** were synthesized (Figure 22). All the synthesized compounds were screened for anticonvulsant activity by maximal electroshock (MES) convulsion method. Phenobarbitone sodium and lamotrigine were used as reference drugs and % reduction of time spent by animals in extension, flexion, clonus, and stupor phase were noted. Compounds** 22d** and** 22f** showed more potent anticonvulsant activity when compared with that of the standard drug [49].

### 2.5. Anticancer Activity

**Figure 23:**
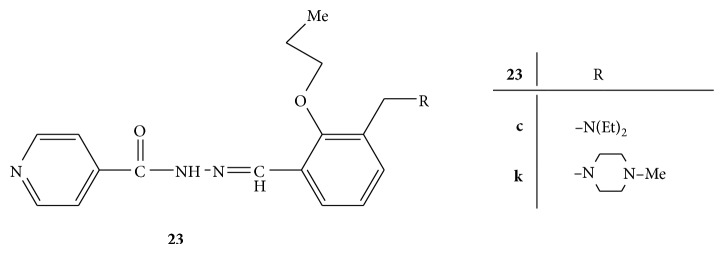


A series of novel Mannich bases of 2-propoxybenzylideneisonicotinohydrazide,** 23** (Figure 23), were screened for the cytotoxicity studies against the A549 human lung adenocarcinoma. Dulbecco's modified eagle medium was used for the growth of cells and supplemented with glutamine, fetal bovine serum, penicillin, and streptomycin. Gemcitabine was used as standard drug. The viability of the cells was assessed by MTT assay. Cells were placed in 96-well plate and after 24 h they were treated with different test compounds. In each well MTT in phosphate buffer saline was added and incubated and then DMSO was added to each well and kept in incubator. From all the compounds synthesized,** 23c** and** 23k** exhibited potential cytotoxic activity superior to that of the standard drug, gemcitabine [50].

**Figure 24:**
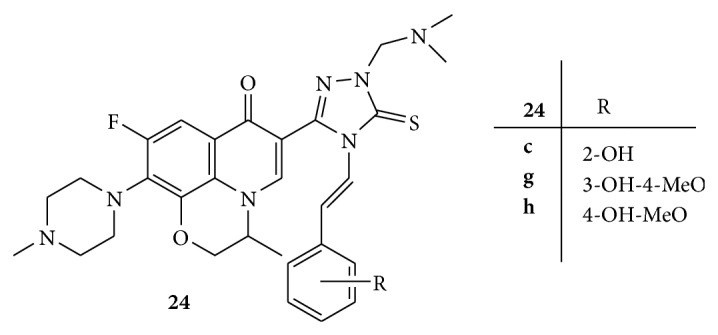


Schiff-Mannich bases of fluoroquinolones,** 24** (Figure 24), from antibacterial analogs were synthesized and screened for antitumor activity against L1210, CHO, and HL60 cell lines. The cell lines were maintained in RPMI 1640 medium supplemented with fetal bovine serum. The medium containing 5 × 10^3^ cells was seeded into 96-well microplate and compounds to be tested were then added. Plates were incubated and MTT solution in PBS was added to each well. The plates were further incubated for 4 h and DMSO was added to the wells containing CHO cell lines. Dodecylbenzenesulfonate (10%) was added to cells containing L1210 and HL60. The optimal density of each well was measured at 570 nm. Among the synthesized compounds** (24a–24k)**,** 24c**,** 24g**, and** 24h** exhibited significant cytotoxic activity than the parent compound [51].

**Figure 25:**
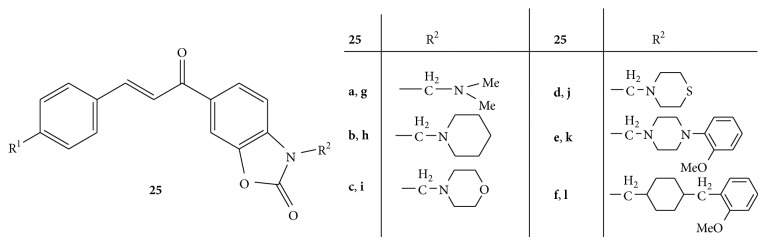


Twelve new Mannich bases of 6-(3-aryl-2-propenoyl)-2(3H)-benzoxazolones** 25** (Figure 25) were synthesized and evaluated for cytotoxicity in human pre-B-cell leukemia cell line BV-173 using MTT-dye reduction assay. The selected compounds were further evaluated on chronic myeloid leukemia K-562 cells. The cytotoxic studies revealed that the compound bearing 4-methoxy function** (25g–l)** at R^1^ in B-ring causes significant decrease in potency while nonmethoxy substituted analogues** (25a–f)** bearing bulky basic substituent at nitrogen were found to be more potent [52].

**Figure 26:**
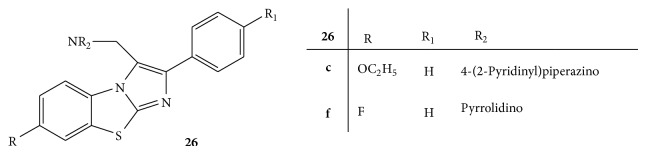


Some novel Mannich bases of 2-arylimidazo[2,1-b]benzothiazoles** 26(a–h)** (Figure 26) were synthesized and evaluated for their anticancer activity. The anticancer activity was studied on HepG2 (hepatocarcinoma cell line), MCF-7 (breast carcinoma cell line), and HeLa 9 (human cervical carcinoma cell line) cell lines using MTT assay. All the synthesized compounds have shown cytotoxic activity against the used cell lines. The compounds** 26c** and** 26f** substituted with 4-(2-pyridinyl)piperazino and pyrrolidino, respectively, were found to induce G2/M cell cycle arrest with downregulation of cyclin B and upregulation of Chk2 protein. Both compounds have shown characteristic features of apoptosis. The compound** 26f** could be considered the potential lead for its development as a novel anticancer agent [53].

### 2.6. Antioxidant Activity

**Figure 27:**
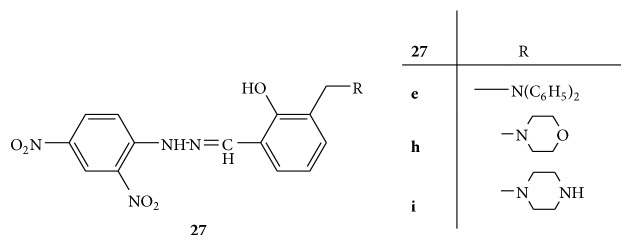


Ten new Mannich bases were synthesized using (E)-2-{[-2-(2,4-dinitrophenyl)hydrazono]methyl}phenol,** 27** (Figure 27), as a key intermediate and screened for antioxidant activity. From all the synthesized derivatives** 27(a–j)**, the compound** 27h** containing morpholine moiety was found to be most active followed by the compound** 27i** with piperazine moiety and compound** 27e** with diphenylamine moiety [54].

**Figure 28:**
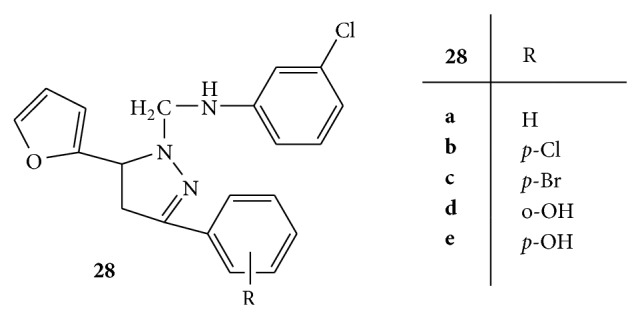


A novel series of Mannich bases of pyrazolines** 28(a–e)** was synthesized (Figure 28) and evaluated for the antioxidant activity using DPPH radical and NO radical scavenging methods. Ascorbic acid and rutin were employed as standard drugs for comparison. The results of both the assays suggested that compounds** 28d** and** 28e** have shown highest scavenging activity. The compound** 28e** was found to have highest antioxidant capacity as compared to both of the standard drugs. These results confirmed that phenolic compounds possess high antioxidant ability as compared to nonphenolic compounds [55].

**Figure 29:**
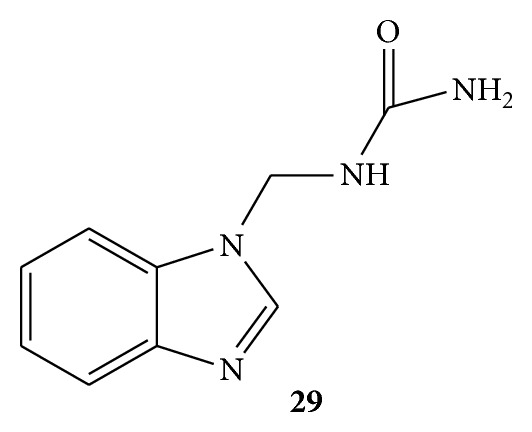


**Figure 30:**
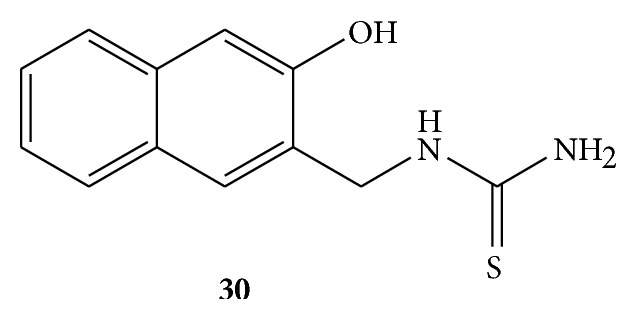


Two Mannich bases of benzamide, that is, 1-((1H-benzod]imidazole-1-yl)methyl)urea (BIUF)** 29** (Figure 29) and 1-((3-hydroxynaphthalen-2-yl)methyl)thiourea (TNTUF)** 30** (Figure 30) were synthesized and evaluated for the antioxidant activity. Hydrogen peroxide radical scavenging, DPPH radical scavenging, and reducing power assays were used for antioxidant activity estimation. Ascorbic acid was used as standard drug. Both of the Mannich bases were found to be active antioxidant agents due to presence of electron releasing amide group in them. BIUF was found to more active than TNTUF due to the presence of two N atoms in the benzimidazole adjoined with amide group [56].

**Figure 31:**
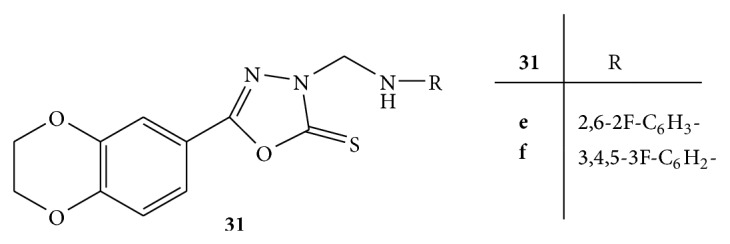


A series of novel Mannich bases of 1,3,4-oxadiazole derivatives having 1,4-benzodioxan** 31** (Figure 31) were synthesized. All the synthesized novel compounds were screened for their* in vitro* antioxidant activity by using 2,2′-diphenyl-1-picrylhydrazyl radical (DPPH), 2,2′-azinobis (3-ethylbenzothiazoline-6-sulfonate) cationic radical (ABTS^∗+^), and ferric reducing antioxidant power (FRAP) scavenging assays [57].

All the compounds exhibited good antioxidant activities. BHT and Trolox were used as standard compounds for the comparison. The compounds** 31e** and** 31f** with multifluoro substitution on benzene ring showed significant radical scavenging ability in all three scavenging assays [57].

### 2.7. Analgesic Activity

**Figure 32:**
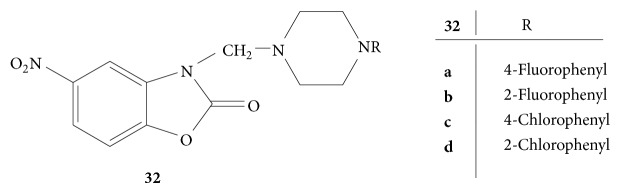


A novel series of Mannich bases of 5-nitro 3-substituted piperazino methyl-2-benzoxazolinones,** 32(a–d)** (Figure 32), were prepared and studied for the analgesic activity by* p*-benzoquinone induced writhing test. Among all the synthesized derivatives the compounds bearing electron withdrawing groups at* para* position such as fluoro and chloro showed potent analgesic activity [9].

**Figure 33:**
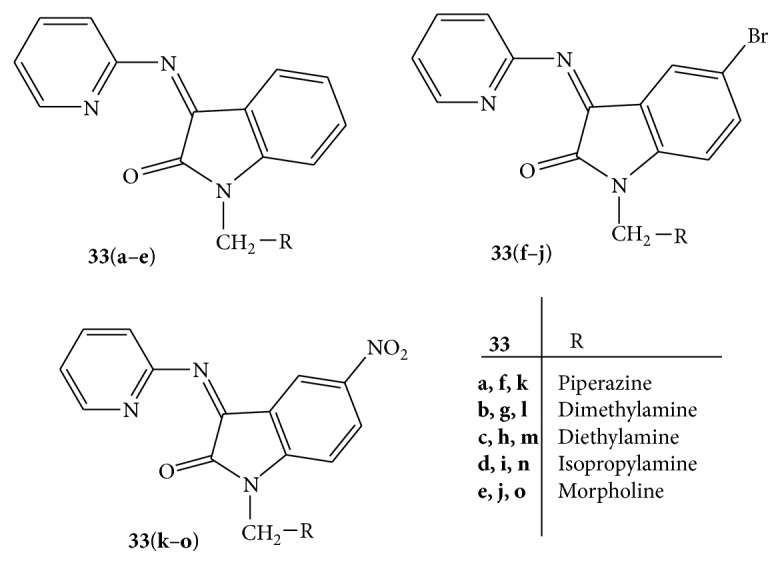


Mannich bases of various 2-amino pyridine,** 33** (Figure 33), derivatives were synthesized and screened for analgesic activity using acetic acid induced writhing test and hot plate test. A series of Mannich bases were prepared by condensing 2-amino pyridine with isatin and treating the above condensed products with various secondary amines. The analgesic activities were evaluated using central and peripheral analgesic assay. Indomethacin, diclofenac, and morphine were used as reference drugs. Among the various synthesized compounds** 33e**,** 33g**,** 33h**,** 33j**,** 33k**, and** 33l** revealed potent analgesic activity. Compounds** 33f** and** 33g** were found to be most potent against central and peripheral analgesic assay, respectively [58].

### 2.8. Antimycobacterial Activity

**Figure 34:**
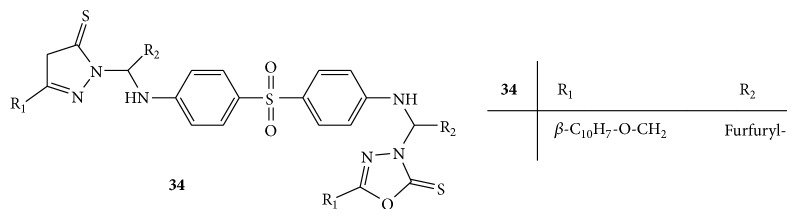


By reacting oxadiazole derivatives, dapsone, and suitable aldehyde in the presence of methanol, a series of new oxadiazole Mannich bases** 34(a–n)** (Figure 34) was synthesized. The synthesized Mannich bases were evaluated for their antimycobacterial activity against* M. tuberculosis* H37Rv and INH resistant* M. tuberculosis* using agar dilution method. Among all the synthesized compounds, compound** 34d** 3-{2-furyl[4-(4-{2-furyl[5-(2-naphthyloxymethyl)-2-thioxo-2,3-dihydro-1,3,4-oxadiazol-3-yl]methylamino}phenylsulfonyl)-anilino]methyl}-5-(2-naphthyloxymethyl)-2,3-dihydro-1,3,4-oxadiazole-2-thione was found to be the most promising compound active against* M. tuberculosis* H37Rv and isoniazid (INH) resistant* M. tuberculosis* with minimum inhibitory concentration (MIC) of 0.1 *μ*M and 1.10 *μ*M, respectively [59].

**Figure 35:**
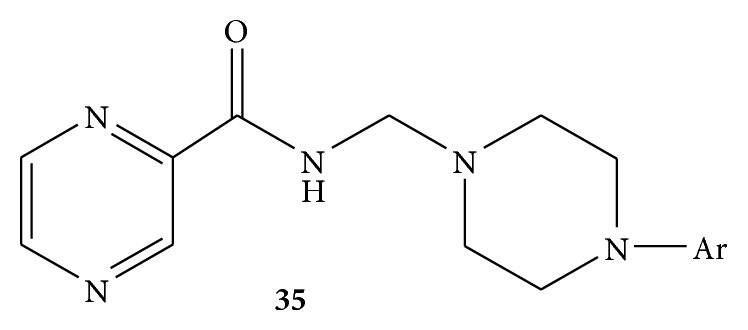


By using microwave irradiation method, a series of Mannich bases of pyrazinamide was synthesized. The synthesized compounds were evaluated for their* in vitro* and* in vivo* antimycobacterial activity. For* in vivo* activity* M. tuberculosis* H37Rv (MTB) strain was used. Among all the synthesized compounds, 1-cyclopropyl-6-fluoro-1,4-dihydro-8-methoxy-7-(3-methyl-4-((pyrazine-2-carboxamido)methyl)piperazin-1-yl)-4-oxoquinoline-3 carboxylic acid** 35** (Figure 35) was found to be the most active compound* in vitro* with MIC of 0.39 and 0.2 lg/mL against MTB and multidrug-resistant MTB, respectively. In the* in vivo* animal model 17 decreased the bacterial load in lung and spleen tissues with 1.86 and 1.66 − log⁡⁡10 protections, respectively [60].

A series of Mannich bases of 3-[*p*-(5-arylpyrazolin-3-yl) phenyl]syndone** 36(a–j)** (Figure 36) was synthesized and studied for the antimycobacterial activity. The antimycobacterial activity of the test compounds was evaluated against the standard strain of* M. tuberculosis* H37Rv. Streptomycin and pyrazinamide were used as standards. The results exposed the fact that the compounds** 36c**,** 36d**,** 36e**,** 36g**, and** 36i** exhibited good activity. The promising activities are accredited to the presence of long alkylating chains with electron donating groups like hydroxyl, amino, methylene, and ethylene through mesomeric effect attached to the pyrazoline moiety [61].

## 3. Conclusion

As demonstrated by the frame of work reviewed in this paper, Mannich bases and their derivatives are found to have potent diverse activities. This review summarized various biological activities of Mannich base derivatives in present scenario. It can be concluded that Mannich bases have remarkable biological potential which is remaining unexplored. However this review would expectantly shed light on ways to raise the therapeutic worth and specificity of Mannich bases. This bioactive core has maintained the interest of researchers in gaining the most suggestive and conclusive access in the field of various Mannich bases of medicinal importance from last decades and also endorsed the researchers for design of novel heterocyclic/aryl derivatives for progress of new environment-friendly technology.

## Figures and Tables

**Figure 36:**